# Community case management of fast-breathing pneumonia with 3 days oral amoxicillin vs 5 days cotrimoxazole in children 2-59 months of age in rural Pakistan: A cluster randomized trial

**DOI:** 10.7189/jogh.12.04097

**Published:** 2022-12-29

**Authors:** Sheraz Ahmed, Shabina Ariff, Sajid Muhammed, Arjumand Rizvi, Imran Ahmed, Sajid Bashir Soofi, Zulfiqar A Bhutta

**Affiliations:** 1 Department of Pediatrics and Child Health, The Aga Khan University, Karachi, Sindh, Pakistan; 2 Center of Excellence in Women and Child Health, The Aga Khan University, Karachi, Sindh, Pakistan; 3 Centre for Global Child Health, The Hospital for Sick Children, Toronto, Ontario, Canada

## Abstract

**Background:**

Pneumonia is the leading cause of mortality in under-five children and most of these deaths occur in South-East Asia and Africa. Fast breathing pneumonia if not treated can progress to lower chest indrawing pneumonia. Treatment recommendation by the World Health Organization (WHO) for fast-breathing pneumonia includes oral amoxicillin and cotrimoxazole (as an alternative). Due to limited access to health care facilities and skilled health care workers, many children are unable to receive antibiotics. Algorithm-based community case management of pneumonia through trained community health workers has resulted in a decline in morbidity and mortality in low- and middle-income countries (LMIC).

**Methods:**

It was a cluster-randomized, unblinded, community-based trial conducted in the Matiari district of Sindh province, Pakistan. Lady Health Workers (LHWs) were trained in assessing, classifying, and managing fast-breathing pneumonia cases (Respiratory rate of >50 breaths/min) at home with oral amoxicillin for three days and with co-trimoxazole for five days in the intervention and control arms respectively. Children with fast-breathing pneumonia were screened by LHWs and were validated by the study by Community Health Workers (CHWs) within 48 hours. They were followed by the LHWs on days 2, 4, and 14 in intervention and on days 2, 6, and 14 in the control arm. Primary treatment failure was assessed on day 4 in intervention and day 6 in the control arm. A severe pneumonia trial was registered with ClinicalTrials.gov, number NCT01192789.

**Results:**

From February 2008 to March 2010, a total of 5876 children were enrolled by Lady Health Workers as fast breathing pneumonia. On validation visits of the CHWs, 728 (12%) children were excluded. A total of 4984 children were analysed as per protocol: 2480 in intervention and 2504 in control. There were 72 (2.9%) primary treatment failures in the intervention arm as compared to 102 (4%) in the control arm with a risk difference of -0.94 (-2.84%, 0.96%). Secondary treatment failures were almost equal in both arms (4 vs 7 cases). No deaths or serious adverse events were recorded.

**Conclusions:**

This study shows that amoxicillin can be as effective as cotrimoxazole to treat fast-breathing pneumonia cases at the domiciliary level.

**Registration:**

NCT01192789

About eight million deaths annually are attributed to pneumonia in under-five children which makes pneumonia the highest contributor (15%) in under-five child deaths [1]. Although there has been a decrease in pneumonia incidence by 34% and deaths by 63% since 1990, the majority of these deaths still occur in low and middle-income countries with most occurring outside hospital settings [2]. In 2015, the global estimate of pneumonia episodes was 138 million [3]. Pakistan is among five countries that share the burden of 52% of total pneumonia episodes and 49% of pneumonia deaths [1]. Annually, there have been 58 000 under-five children with pneumonia deaths in Pakistan [4].

As per Pakistan’s Demographic and Health Survey conducted in 2012 and 2017, the number of children seeking care at health facilities for Acute Respiratory Infection (ARI) symptoms increased from 64% to 82% [5,6]. Poorly equipped health facilities and lack of health care providers along with poverty lead to deaths in children with pneumonia [7]. About 70% of mortality can be reduced with the timely provision of community case management of pneumonia by trained CHWs in 0-5 years of children [8]. There is also evidence that care-seeking toward lady health workers in Pakistan is very low due to poor community trust in LHWs’ skills and the non-availability of antibiotics with the LHWs [9,10].

Fast breathing pneumonia (Respiratory rate; ≥50 breaths per minute in children aged 2-59; (**Table 1**), if not treated can progress to lower chest indrawing pneumonia. Treatment of fast-breathing pneumonia includes an oral antibiotic.

**Table 1:** Inclusion and exclusion criteria

Inclusion criteria	Exclusion criteria
Age 2 to 59 months	Very severe disease
Presenting to LHWs with fast breathing pneumonia	Persistent vomiting
Informed consent given by a legal guardian	Parent/caretaker refuses to participate
	Currently being treated for non-severe pneumonia with antibiotics
	Suspected or known kerosene oil ingestion
	Audible wheeze
	Children with known asthma
	Children with severe malnutrition

LHWs – lady health workers

The choice of antibiotics as per World Health Organization (WHO) is oral amoxicillin in outpatient settings with low HIV prevalence and in cases where oral amoxicillin is not available, oral co-trimoxazole is an alternative [11].

Pakistan Multicenter Amoxycillin Short Course Therapy (MASCOT) and INDIACLEN Short Course Amoxicillin Pneumonia (ISCAP) Study Groups data had shown that 3 days of oral amoxicillin was equivalent to 5 days of oral amoxicillin for treating fast breathing pneumonia in health facilities [12,13]. WHO used the evidence to recommend 3 days of oral amoxicillin for fast-breathing pneumonia in low-HIV settings for health facilities. No data existed that showed whether LHWs could also treat fast-breathing pneumonia with 3 days of oral amoxicillin at the time of conduct of this study. At that time, the LHW Program of Pakistan recommended 5 days of oral cotrimoxazole. We conducted a trial to test 5 days of oral amoxicillin for lower chest indrawing pneumonia by LHWs [14], so it made sense to test whether LHWs could also treat fast-breathing pneumonia with 3 days of oral amoxicillin.

## METHODS

This study was conducted in the rural district of Matiari, Sindh, Pakistan. It is situated 185 km north of Karachi city. Matiari district has 18 union councils (the smallest administrative unit) with about 1600 villages and a population of approximately 0.7 million [15]. The majority of the adult population is associated with the agriculture-related business. The literacy rate is low and less than half of the population is poor [16]. Several large community-based projects have been conducted in Matiari on maternal, newborn, and child health [17-21]. Data from this study was collected as part of a lower chest indrawing pneumonia study that has been published elsewhere [14]. Initially, it was planned to enrol only lower chest indrawing pneumonia cases but later it was decided to include the fast-breathing pneumonia cases due to ethical obligations.

### Ethical considerations

The study was approved by the Aga Khan Ethical Review Committee and by the Boston University Institutional Review Board.

### Study design

It was a cluster-randomized, un-blinded trial in 18 union councils of Matiari. The cluster was defined as a union council. Clusters were randomized into intervention and control arms through a restricted randomization scheme based on socio-economic characteristics, maternal and child mortality indicators, the number of LHWs, and the population from baseline census data conducted in the study area. Inclusion and exclusion criteria were presented in **Table 1**.

### Sample size, randomization and study procedures

As this was a nested study within a chest indrawing pneumonia study, the sample size and randomization and study procedures have already been described in detail [14].

### Intervention arm

Children with fast breathing pneumonia in the intervention arm received oral amoxicillin of 250mg/5ml for 3 days by LHWs. The dose for 2-11 months was 1 ¼ spoon twice daily and for 12-59 months was ¾ spoon twice daily. Follow-up assessments of cases were done at 2, 4 and 14 days. Pneumonia cases notified by LHWs were validated within 48 hours (day 1 or day 2) by the project-hired CHWs. If there was a mismatch in diagnosis between CHW and LHW, then the study physician validated the cases. Mothers were counselled at each visit to seek care from the nearest health facility in case the child’s condition deteriorates or develops danger signs.

### Control arm

In the control arm, fast-breathing pneumonia cases were treated by oral co-trimoxazole (Sulfamethoxazole 200 mg + trimethoprim 40 mg per 5 ml) for 5 days. Cotrimoxazole dose for 2-6 months of children was half teaspoon twice a day and from 6-59 months was 1 teaspoon twice a day. Follow-up assessments by LHWs were done on day 2, 6, and 14.

### Treatment failure cases

The primary endpoint was treatment failure after enrolment to day 4 (intervention arm) and day 6 (control arm). The secondary endpoint was secondary treatment failure or relapse between day 5 to day 14 (intervention arm) and day 7 to day 14 (control arm) (**Table 2**).

**Table 2:** Definition of treatment failure

Enrolment day	Treatment failure criteria
**Primary treatment failure**	
Day 2	Appearance of any danger sign **or** change of antibiotic without objective criteria of treatment failure **or** appearance of lower chest in drawing
Day 4 (intervention) or day 6 (control)	Appearance of any danger sign **or** fever (≥100 F) **or** appearance of lower chest in drawing **or** fast breathing ≥50 breaths/min **or** change of antibiotic without objective criteria of treatment failure
**Secondary treatment failure or relapse**	
Day 5-14 (intervention) day 7-14 (control)	Lower chest indrawing **or** fever (≥100 F) **or** fast breathing ≥50 breaths/min **or** appearance of any danger sign

Treatment failure cases in fast-breathing pneumonia cases in control and intervention arms were provided with a facilitated referral to the nearest public sector health facility for injectable antibiotics.

## RESULTS

**Figure 1:**
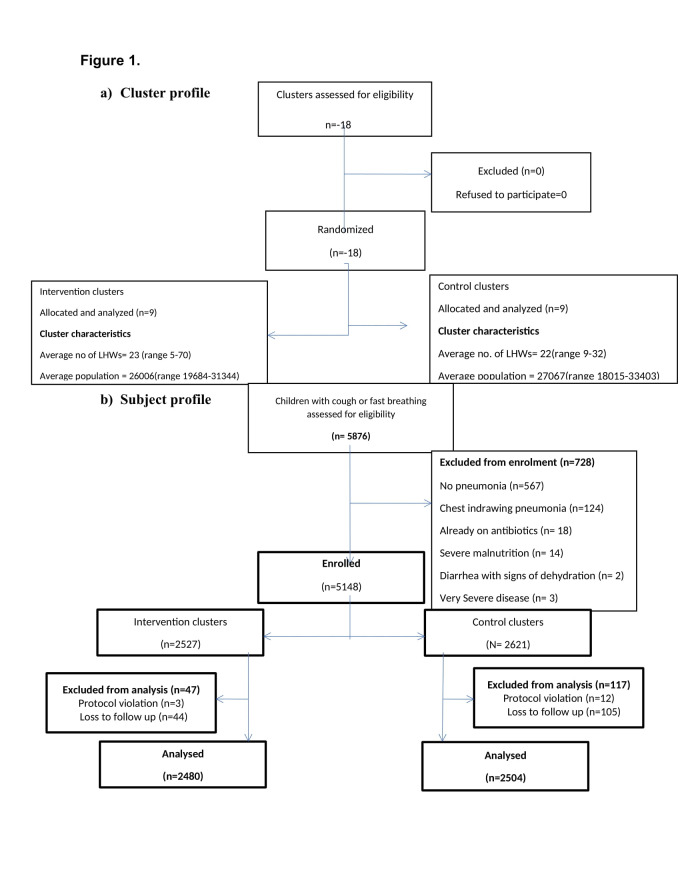
Trial profile.

Nine union councils (UCs) were randomized to the intervention arm and nine to the control arm. The average number of LHWs per UC was 23 (range = 5-70) in the intervention arm vs 22 (range = 9-32) in the control arm. The average population served per UC was 26 006 (range = 19 684-31 344) vs 27 067(range = 18 015-33 403) in intervention and control arms respectively (**Figure 1**).

From February 2008 to March 2012, a total of 5876 children were enrolled by LHWs as cases of fast-breathing pneumonia. On validation visits of the CHWs within 48 hours, 728 (12%) cases were excluded. The majority of cases excluded were of No pneumonia (567) followed by 124 cases of chest indrawing pneumonia and those cases were followed and managed as fast breathing cases. A slightly higher number of cases were enrolled in control (n = 2621) than intervention (n = 2527). 164 cases were not included in the final analyses due to loss to follow-up (n = 149) and protocol violation (n = 15). Per protocol analysis was done. A total of 4984 cases were analysed: 2480 in intervention and 2504 in control (**Figure 1**).

### Baseline characteristics:

In baseline characteristics of intervention and control cases, more males (51%) were recruited. In under 11 months, 783 control cases were enrolled as compared to 597 in the intervention arm. The median age in months was 18 in the control and 26 in the intervention. In the history of current illness, 99% of children had a cough in both groups. Difficult breathing was more reported in the intervention arm than in the control arm. Fast breathing was reported almost as equal, and fever was reported more in the control arm. On the assessment of LHWs, the median respiratory rate was 56 in the intervention vs 57 in the control arm. The median temperature (100°F) was comparable between the groups. The median interquartile range (IQR) weight to age WHO Z-score was high in the intervention -1.01 (-1.77, -0.33) than in the control arm -1.21 (-2.15, -0.41). Median (IQR) enrolment per cluster was 217 (74-429) in intervention and 184 (148-263) in control arms (**Table 3**).

**Table 3:** Baseline characteristics of fast breathing pneumonia cases

	Intervention (n = 2480)	Control (n = 2504)
	**n (%)**	**n (%)**
**Gender**		
Male	1304 (52.6)	1242 (49.6)
Female	1176 (47.4)	1262 (50.4)
**Age group (months)**		
Under 6	249 (10.0)	302 (12.1)
6-11	348 (14.0)	481 (19.2)
12-59	1883 (75.9)	1721 (68.7)
Median age months (IQR)	26 (13-41)	18 (8-28)
**History of current illness reported by caregiver**		
Cough	2472 (99.7)	2497 (99.8)
Difficult breathing	1750 (71.3)	1325 (53.3)
Fast breathing	2428 (98.2)	2437 (97.5)
Fever	1904 (77.1)	2014 (80.7)
**Day 1. Examination by lady health worker**		
Median (IQR) respiratory rate (one-minute count)	54 (53-57)	57 (55-61)
Fast breathing*	2435 (98.3)	2482 (99.3)
Very fast breathing†	34 (1.4)	30 (1.2)
Median (IQR) temperature (°F)	100 (99-100)	100 (99-100)
Median WHO weight for age Z-score (IQR*)	-1.01 (-1.77, 0.33)	-1.21 (-2.15, 0.41)
Median enrolment per cluster (IQR)	217 (74-429)	184 (148-263)

IQR – interquartile range

*Fast breathing – RR> = 50/min.

†Very fast breathing – RR> = 70/min.

### Clinical outcomes

There were 72 (2.9%) primary treatment failure cases in the intervention arm as compared to 102 (4%) cases in the control arm with a risk difference of -0.94 (-2.84%, 0.96%). Confidence intervals were adjusted for clustering using a generalized estimating equation and an exchangeable correlation matrix. Persistent fast breathing (RR>50) on day 4 contributed significantly (51 cases) in treatment failure in intervention while lower chest indrawing on day 6 contributes a similar number of cases ie, 51 in the control arm. Fever contributed equally (0.5%) in both arms while a change of treatment cases was higher (39) in the control arm. Secondary treatment failure cases were almost equal in both arms (4 vs 7 cases) (**Table 4**).

**Table 4:** Cluster-adjusted treatment failure by day 4 (primary outcome intervention) and day 6 (primary outcome control) in children with fast-breathing pneumonia in the intervention and control clusters*

Reason for failure	Intervention	Control	Risk difference (95% CI)^‡^
	**n/n (%)**	**n/n (%)**	
All treatment failure^†^	72/2480 (2.9)	102/2504 (4.1)	-0.94 (-2.84, 0.96)
Reasons for failure			
Inability to drink	1/2480 (0.0)	1/2504 (0.0)	0.01 (-0.09, 0.11)
Abnormally sleepy	0/2480 (0.0)	1/2504 (0.0)	-
Vomit everything	0/2480 (0.0)	1/2504 (0.0)	-
Convulsion	0/2480 (0.0)	1/2504 (0.0)	-
RR≥50/min	51/2480 (2.1)	16/2504 (0.6)	1.74 (0.57, 2.90)
Lower chest indrawing	19/2478 (0.8)	51/2504 (2.0)	-1.20 (-2.03, -0.38)
Fever(temp >100°F)	13/2470 (0.5)	14/2498 (0.6)	-0.10 (-0.58, 0.38)
Change of treatment	7/2480 (0.3)	39/2504 (1.6)	-1.27 (-1.73, -0.82)

CI – confidence interval

*For treatment failure presence of danger signs and change of treatment assessed on day 2 to 4 for intervention and day 2 to 6 for the control group.

†Total failures are not equal to the sum of the individual failure reasons as subjects could fail for more than one reason.

‡Confidence intervals are adjusted for clustering using a generalized estimating equation and an exchangeable correlation matrix.

We also analysed the treatment failure in the intervention arm on day 6 to see if natural disease progression was different in the intervention arm as amoxicillin was provided for 3 days. As per **Table 5**, the primary treatment failure in the intervention arm was reduced to 62 (2.5%) on day 6 with a risk difference of -1.51 (-3.21, 0.18). Treatment failure due to persistent fast breathing reduced from 51 to 38 cases from day 4 to day 6. Treatment failure due to lower chest indrawing increased from 19 to 25 cases from day 4 to day 6.

**Table 5:** Cluster-adjusted treatment failure by day 6 (primary outcome intervention) and day 6 (primary outcome control) in children with fast-breathing pneumonia in the Intervention and control clusters*

Reason for failure	Intervention	Control	Risk difference (95% CI)^‡^
	**n/n (%)**	**n/n (%)**	
All treatment failure^†^	62/2480 (2.5)	102/2504 (4.1)	-1.51 (-3.21, 0.18)
Reasons for failure			
Inability to drink	1/2480 (0.0)	1/2504 (0.0)	0.01 (-0.09, 0.11)
Abnormally sleepy	0/2480 (0.0)	1/2504 (0.0)	-
Vomit everything	0/2480 (0.0)	1/2504 (0.0)	-
Convulsion	0/2480 (0.0)	1/2504 (0.0)	-
RR≥50/min	38/2480 (1.5)	16/2504 (0.6)	1.00 (0.00-1.99)
Lower chest indrawing	25/2478 (1.0)	51/2504 (2.0)	-0.91 (-1.88, 0.06)
Fever (temp >100°F)	17/2470 (0.7)	14/2498 (0.6)	-0.04 (-0.63, 0.55)
Change of treatment	10/2480 (0.4)	39/2504 (1.6)	-1.16 (-1.47, -0.85)

CI – confidence interval

*For treatment failure presence of danger signs and change of treatment assessed on day 2 to 6 for intervention and control group.

†Total failures is not equal to the sum of the individual failure reasons as subjects could fail for more than one reason.

‡Confidence intervals are adjusted for clustering using a generalized estimating equation and an exchangeable correlation matrix.

As there were differences in age and baseline symptoms (eg, fever 71% vs 53%) between the two arms. We adjusted the risk difference of treatment failure by age, gender, fever, and difficulty breathing to account for the confounding effect. The estimates remained almost the same (data not shown).

## DISCUSSION

The results of the study showed that there was no difference in treatment failure rate with either oral amoxicillin or cotrimoxazole if fast-breathing pneumonia cases were managed at home by LHWs. The treatment failure rate with oral amoxicillin and cotrimoxazole was low in this study as compared to some previous studies [12,13,22-27]. The treatment failure rate in other studies for amoxicillin ranged 8%-23% and for cotrimoxazole ranged from 9.5%-39%. The only exceptions are two studies, one study conducted in Brazil by the PNEUMOPAC-Efficacy Study Group which treated fast breathing pneumonia cases with oral amoxicillin and the failure rate was 3% [27] and In the Haripur community-based study, treatment failure for amoxicillin was 3.6% and for cotrimoxazole was 9.1% [28] There are other two community-based studies in young infants (<60 days) conducted recently in which treatment failure rate in amoxicillin was comparable with this study, one study was conducted in Pakistan in which the treatment failure rate with 3-day amoxicillin was 2.6% vs 4.9% in the placebo group [29]. Similarly, in the multicountry trial conducted in rural Bangladesh, Ethiopia, India, and Malawi, the treatment failure with 7-day treatment with oral amoxicillin was 5.4% [30].

The difference in the treatment failure rate could be due to the majority of previous trials being in hospital settings to compare the effectiveness of amoxicillin and cotrimoxazole or to compare the different doses and duration of amoxicillin and cotrimoxazole in treating fast-breathing pneumonia cases. Another possible reason for the difference in treatment failure could be a single respiratory rate cut-off used in this study. As far as our knowledge is concerned, it was one of the first community-based trials for fast-breathing pneumonia along with a similar trial conducted at Haripur, Pakistan site at the same time with the same collaborating partners, trial design, inclusion, and exclusion criteria [28]. Almost all studies reported male dominance in fast-breathing pneumonia enrolment [12,13,22-24].

The majority of primary treatment failure in the control arm was due to lower chest indrawing compared to fast breathing in the intervention arm. We don’t know the exact reason(s) but it could be due to the children being younger and underweight in the control arm as compared to the intervention arm. A low number of lower chest indrawing as treatment failure caused in the intervention arm shows that amoxicillin might have prevented some progression of disease from non-severe to severe pneumonia. Persistent fast breathing cases in the intervention arm were reduced on day 6. No death was reported in our trial. This could be due to regular follow-ups of LHWs as treatment failure cases were referred promptly to nearby health facilities for further management. It shows that pneumonia mortality can be reduced if the cases are diagnosed and treated promptly and followed regularly along with the counselling of parents in recognition of danger signs [28,30]. Very few adverse events were noted with oral amoxicillin.

Although treatment with amoxicillin is costlier than cotrimoxazole short-term therapy with amoxicillin offers better compliance and less antibiotic resistance [13] and the Pakistani population is more resistant to cotrimoxazole [31], so three days of amoxicillin treatment offer more benefits. There is evidence that following the WHO pneumonia case management algorithm promoted the rational use of antibiotics in children [32,33].

As this study was done by public sector community-based workers, so it is easy for policymakers to scale it across Pakistan to save thousands of under five-year children as pneumonia is the leading cause of death in under-five children in Pakistan [1].

The limitation of this study was that no blood samples or chest radiographs were taken, and the diagnosis was based solely on clinical signs. Fast breathing can also have a viral aetiology and oral amoxicillin may not be effective, but it is difficult to differentiate viral vs bacterial infections clinically. Additionally, the sample size was powered for the severe pneumonia study and not for fast breathing study.

## CONCLUSIONS

This study shows that amoxicillin can be as effective as cotrimoxazole to treat fast-breathing pneumonia cases at the domiciliary level. It also shows that LHWs can treat pneumonia cases at home if provided with proper supervision and training.

## References

[1] Perin J Mulick A Yeung D Villavicencio F Lopez G Strong KL Global, regional, and national causes of under-5 mortality in 2000-19: An updated systematic analysis with implications for the sustainable development goals. Lancet Child Adolesc Health. 2022;6:106-15 10.1016/S2352-4642(21)00311-4 34800370 PMC8786667

[2] Nair H Simoes EA Rudan I Gessner BD Azziz-Baumgartner E Zhang JSF Severe Acute Lower Respiratory Infections Working Group Global and regional burden of hospital admissions for severe acute lower respiratory infections in young children in 2010: A systematic analysis. Lancet. 2013;381:1380-90. 10.1016/S0140-6736(12)61901-1 23369797 PMC3986472

[3] McAllister DA Liu L Shi T Chu Y Reed C Burrows J Global, regional, and national estimates of pneumonia morbidity and mortality in children younger than 5 years between 2000 and 2015: A systematic analysis. Lancet Glob Health. 2019;7:e47-57. 10.1016/S2214-109X(18)30408-X 30497986 PMC6293057

[4] The United Nations Children’s Fund. One child dies of pneumonia every 39 seconds, agencies warn. UNICEF. Available from: www.unicef.org/pakistan/press-releases/one-child-dies-pneumonia-every-39-seconds-agencies-warn. Accessed: 9 April 2022.

[5] National Institute of Population Studies (NIPS) [Pakistan] and ICF. Pakistan Demographic and Health Survey (PDHS) 2017-18. Islamabad, Pakistan, and Rockville, Maryland, USA: NIPS and I CF; 2018 Available from: http://nips.org.pk/abstract_files/PDHS%20-%202017-18%20Key%20indicator%20Report%20Aug%202018.pdf. Accessed: 9 April 2022.

[6] National Institute of Population Studies (NIPS) [Pakistan] and ICF International. Pakistan Demographic and Health Survey (PDHS) 2012-2013. Islamabad: 2013 Available from: http://www.nips.org.pk/abstract_files/PDHS%20Final%20Report%20as%20of%20Jan%2022-2014.pdf. Accessed: 9 April 2020.

[7] Källander K Hildenwall H Waiswa P Galiwango E Peterson S Pariyo G Delayed care seeking for fatal pneumonia in children aged under five years in uganda: A case-series study. Bull World Health Organ. 2008;86:332-8. 10.2471/BLT.07.049353 18545734 PMC2647445

[8] Theodoratou E Al-Jilaihawi S Woodward F Ferguson J Jhass A Balliet M The effect of case management on childhood pneumonia mortality in developing countries. Int J Epidemiol. 2010;39 Suppl 1:i155-71. 10.1093/ije/dyq032 20348118 PMC2845871

[9] Sadruddin S Khan IU Bari A Khan A Ahmad I Qazi SA Effect of community mobilization on appropriate care seeking for pneumonia in haripur, pakistan. J Glob Health. 2015;5:010405. 10.7189/jogh.05.010405 25798232 PMC4357212

[10] Aftab W Shipton L Rabbani F Sangrasi K Perveen S Zahidie A Exploring health care seeking knowledge, perceptions and practices for childhood diarrhea and pneumonia and their context in a rural pakistani community. BMC Health Serv Res. 2018;18:44. 10.1186/s12913-018-2845-z 29374472 PMC5787321

[11] Grant GB Campbell H Dowell SF Graham SM Klugman KP Mulholland EK World Health Organization Department of Child and Adolescent Health and Development Recommendations for treatment of childhood non-severe pneumonia. Lancet Infect Dis. 2009;9:185-96. 10.1016/S1473-3099(09)70044-1 19246022 PMC7172451

[12] Pakistan MASCT (MASCOT) pneumonia study group. Clinical efficacy of 3 days versus 5 days of oral amoxicillin for treatment of childhood pneumonia: A multicentre double-blind trial. Lancet. 2002;360:835-41. 10.1016/S0140-6736(02)09994-4 12243918

[13] Agarwal G Awasthi S Kabra SK Kaul A Singhi S Walter SD ISCAP Study Group Three day versus five day treatment with amoxicillin for non-severe pneumonia in young children: A multicentre randomised controlled trial. BMJ. 2004;328:791. 10.1136/bmj.38049.490255.DE 15070633 PMC383371

[14] Soofi S Ahmed S Fox MP MacLeod WB Thea DM Qazi SA Effectiveness of community case management of severe pneumonia with oral amoxicillin in children aged 2-59 months in matiari district, rural pakistan: A cluster-randomised controlled trial. Lancet. 2012;379:729-37. 10.1016/S0140-6736(11)61714-5 22285055

[15] Pakistan Bureau of Statistics. Matiari district population and household detail: Census 2017. Islamabad: Pakistan Bureau of Statistics; 2018 Available from: http://www.pbscensus.gov.pk/sites/default/files/bwpsr/sindh/MATIARI_BLOCKWISE.pdf. Accessed: 9 April 2020.

[16] Bhutta ZA Soofi S Cousens S Mohammad S Memon ZA Ali I Improvement of perinatal and newborn care in rural pakistan through community-based strategies: A cluster-randomised effectiveness trial. Lancet. 2011;377:403-12. 10.1016/S0140-6736(10)62274-X 21239052

[17] Ahmed S Ariff S Soofi SB Hussain A Hotwani A Yaqoob M Challenges in implementation of the ANISA protocol at the matiari site, pakistan. Pediatr Infect Dis J. 2016;35(5 Suppl 1):S65-9. 10.1097/INF.0000000000001110 27070069

[18] Habib MA Soofi S Sheraz A Bhatti ZS Okayasu H Zaidi SZ Zinc supplementation fails to increase the immunogenicity of oral poliovirus vaccine: A randomized controlled trial. Vaccine. 2015;33:819-25. 10.1016/j.vaccine.2014.12.001 25500307

[19] Saha SK Schrag SJ El Arifeen S Mullany LC Shahidul Islam M Shang N Causes and incidence of community-acquired serious infections among young children in south asia (ANISA): An observational cohort study. Lancet. 2018;392:145-59. 10.1016/S0140-6736(18)31127-9 30025808 PMC6053599

[20] Iqbal NT Syed S Kabir F Jamil Z Akhund T Qureshi S Pathobiome driven gut inflammation in pakistani children with environmental enteric dysfunction. PLoS One. 2019;14:e0221095. 10.1371/journal.pone.0221095 31442248 PMC6707605

[21] Ali A Akhund T Warraich GJ Aziz F Rahman N Umrani FA Respiratory viruses associated with severe pneumonia in children under 2 years old in a rural community in pakistan. J Med Virol. 2016;88:1882-90. 10.1002/jmv.24557 27096404 PMC7166621

[22] Vilas-Boas AL Fontoura MS Xavier-Souza G Araujo-Neto CA Andrade SC Brim RV PNEUMOPAC-Efficacy Study Group Comparison of oral amoxicillin given thrice or twice daily to children between 2 and 59 months old with non-severe pneumonia: A randomized controlled trial. J Antimicrob Chemother. 2014;69:1954-9. 10.1093/jac/dku070 24648506

[23] Rajesh SM Singhal V Clinical effectiveness of co-trimoxazole vs. amoxicillin in the treatment of non-severe pneumonia in children in india: A randomized controlled trial. Int J Prev Med. 2013;4:1162-8. 24319556 PMC3843303

[24] Rasmussen ZA Bari A Qazi S Rehman G Azam I Khan S (Cotrimoxazole Multicentre Efficacy) Study Group. Randomized controlled trial of standard versus double dose cotrimoxazole for childhood pneumonia in pakistan. Bull World Health Organ. 2005;83:10-9. 15682244 PMC2623470

[25] Catchup Study Group Clinical efficacy of co-trimoxazole versus amoxicillin twice daily for treatment of pneumonia: A randomised controlled clinical trial in pakistan. Arch Dis Child. 2002;86:113-8. 10.1136/adc.86.2.113 11827905 PMC1761064

[26] Straus WL Qazi SA Kundi Z Nomani NK Schwartz B Antimicrobial resistance and clinical effectiveness of co-trimoxazole versus amoxycillin for pneumonia among children in pakistan: Randomised controlled trial. pakistan co-trimoxazole study group. Lancet. 1998;352:270-4. 10.1016/S0140-6736(97)10294-X 9690406

[27] Fontoura MS Araujo-Neto CA Andrade SC Brim RV Matutino AR Silva CC PNEUMOPAC-Efficacy Study Group Clinical failure among children with nonsevere community-acquired pneumonia treated with amoxicillin. Expert Opin Pharmacother. 2010;11:1451-8. 10.1517/14656561003777034 20408745

[28] Sadruddin S Khan IUH Fox MP Bari A Khan A Thea DM Comparison of 3 days amoxicillin versus 5 days co-trimoxazole for treatment of fast-breathing pneumonia by community health workers in children aged 2-59 months in pakistan: A cluster-randomized trial. Clin Infect Dis. 2019;69:397-404. 10.1093/cid/ciy918 30596964 PMC6637273

[29] Tikmani SS Muhammad AA Shafiq Y Shah S Kumar N Ahmed I Azam I Pasha O Zaidi AK Ambulatory treatment of fast breathing in young infants aged <60 Days: A Double-Blind, Randomized, Placebo-Controlled Equivalence Trial in Low-Income Settlements of Karachi. Clin Infect Dis. 2017;64:184-9. 10.1093/cid/ciw690 27941119 PMC5853586

[30] Enhanced Management of Pneumonia in Community (EMPIC) Study Nisar YB Community-based amoxicillin treatment for fast breathing pneumonia in young infants 7-59 days old: A cluster randomised trial in rural bangladesh, ethiopia, india and malawi. BMJ Glob Health. 2021;6. 34417274 10.1136/bmjgh-2021-006578 PMC8381301

[31] Straus WL Qazi SA Kundi Z Nomani NK Schwartz B Antimicrobial resistance and clinical effectiveness of co-trimoxazole versus amoxycillin for pneumonia among children in pakistan: Randomised controlled trial. pakistan co-trimoxazole study group. Lancet. 1998;352:270-4. 10.1016/S0140-6736(97)10294-X 9690406

[32] Gouws E Bryce J Habicht JP Amaral J Pariyo G Schellenberg JA Improving antimicrobial use among health workers in first-level facilities: Results from the multi-country evaluation of the integrated management of childhood illness strategy. Bull World Health Organ. 2004;82:509-15. 15508195 PMC2622903

[33] Qazi SA Rehman GN Khan MA Standard management of acute respiratory infections in a children’s hospital in pakistan: Impact on antibiotic use and case fatality. Bull World Health Organ. 1996;74:501-7. 9002330 PMC2486861

